# Smoking and Risk of Atrial Fibrillation Recurrence After Catheter Ablation: A Meta‐Analysis and Meta‐Regression

**DOI:** 10.1002/joa3.70452

**Published:** 2026-08-24

**Authors:** Frengki Prabowo Saputro Wijayanto, Artha Maressa Theodora Simanjuntak, Muhammad Reva Aditya, Alifian Arkan Zaky Kusnadi, Alphatar Magnus Jonathan Tambunan, Harmony Tiffany Clay, Erlangga Dief Putra Hamam, Christopher Daniel Tristan, Raymond Pranata

**Affiliations:** ^1^ Department of Medicine, Faculty of Medicine Public Health and Nursing Universitas Gadjah Mada Yogyakarta Indonesia; ^2^ Department of Medicine, Faculty of Medicine Universitas Brawijaya Malang Indonesia; ^3^ Department of Medicine, Faculty of Medicine Universitas Sebelas Maret Surakarta Indonesia; ^4^ Faculty of Medicine Universitas Pelita Harapan Tangerang Indonesia; ^5^ Department of Cardiovascular Medicine Siloam Hospitals Lippo Village Tangerang Indonesia

**Keywords:** atrial fibrillation, catheter ablation, meta‐analysis, recurrence, smoking

## Abstract

**Background:**

Atrial fibrillation (AF) is the most common sustained cardiac arrhythmia worldwide. Although catheter ablation is an established rhythm‐control strategy, recurrence remains frequent. Smoking is a potentially modifiable risk factor, but its impact on post‐ablation AF recurrence remains uncertain. This study evaluated the association between smoking status and AF recurrence following catheter ablation.

**Method:**

Six databases were searched up to March 2026. Studies including patients with AF undergoing first‐time catheter ablation and reporting recurrence outcomes stratified by smoking status were included. Sensitivity, subgroup, and meta‐regression analyses were performed. Publication bias was evaluated using Egger's test and trim‐and‐fill analysis.

**Result:**

Twenty‐two studies comprising 6047 participants were included. Smoking was associated with a significantly increased risk of AF recurrence following catheter ablation (OR 1.42, 95% CI: 1.09–1.85; *p* = 0.0096), with substantial heterogeneity (*I* ^2^ = 65.8%). Sensitivity analysis confirmed the robustness of the findings (OR 1.35, 95% CI: 1.05–1.73). No significant small‐study effects were detected (Egger's *p* = 0.4280), although trim‐and‐fill analysis attenuated the association to borderline significance. Meta‐regression identified body mass index (BMI) as a significant moderator, with a weaker association observed in populations with higher BMI.

**Conclusion:**

Smoking is associated with a modest but significant increase in the risk of AF recurrence after catheter ablation. Future studies should evaluate the impact of smoking intensity, duration, and cessation on ablation outcomes using patient‐level data.

## Graphical abstract

This meta‐analysis demonstrated that smoking was associated with a higher risk of atrial fibrillation recurrence after catheter ablation (OR 1.42, 95% CI: 1.09–1.85). Among ablation‐modality subgroups, the association was significant for radio Meta‐regression identified BMI as a significant moderator, suggesting that higher BMI attenuated the association between smoking and AF recurrence.

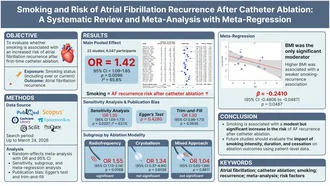

## 1 Introduction

Atrial fibrillation (AF) is the most prevalent sustained cardiac arrhythmia worldwide, characterized by disorganized atrial electrical activity that overrides normal sinus node function. With an estimated global prevalence reaching approximately 58.9 million cases, AF imposes a substantial epidemiological and economic burden on healthcare systems [1, 2]. The economic impact is primarily driven by hospitalizations and the management of severe comorbidities, most notably stroke and heart failure, which exponentially increase healthcare expenditures [3]. Beyond its clinical and economic impact, AF significantly impairs patients' quality of life. Evidence from the DEFINE AFib study demonstrated that experiencing just 6 min of AF daily significantly reduces quality of life, with adverse effects escalating rapidly as the arrhythmia burden increases [4].

Catheter ablation has emerged as a cornerstone first‐line rhythm‐control strategy for symptomatic paroxysmal and persistent AF. Extensive evidence indicates that ablation is superior to antiarrhythmic drug therapy in maintaining sinus rhythm and improving long‐term patient outcomes [5]. However, despite procedural advancements, the long‐term efficacy of catheter ablation remains constrained by a high rate of AF recurrence. Long‐term follow‐up indicates that five‐year recurrence rates reach approximately 32% for paroxysmal AF and climb to over 53% for persistent AF [6]. This high recurrence rate has shifted clinical focus toward identifying and managing pre‐procedural risk factors. These factors are broadly categorized into non‐modifiable traits, such as age and genetics, and modifiable metabolic and lifestyle factors, including hypertension, obesity, obstructive sleep apnea, and tobacco use [7].

Previous systematic reviews have established the prognostic impact of other modifiable factors, such as elevated body mass index and epicardial adipose tissue, on post‐ablation AF recurrence [8, 9]. While smoking is a well‐established pro‐arrhythmic factor known to induce atrial oxidative stress, systemic inflammation, and adverse structural remodeling. evidence regarding its specific impact on ablation outcomes remains conflicting [10, 11, 12, 13, 14, 15]. Several observational studies identify active smoking as a strong independent predictor of recurrence [16], whereas others report no significant association [17, 18]. Reflecting this uncertainty, the 2023 ACC/AHA/ACCP/HRS Guidelines formally acknowledge cigarette smoking as a risk factor for post‐ablation AF recurrence, yet this recommendation is currently based solely on individual cohort studies rather than a robust, quantitative systematic synthesis [19]. A recent large‐scale meta‐analysis by Chan et al. pooled data across 152 studies (*n* = 49 919) to identify multiple clinical risk factors for AF recurrence after ablation; however, smoking as an exposure was evaluated in only three of these studies, comprising 1122 patients, and was not the primary focus of that analysis [20].

To our knowledge, no previous systematic review and meta‐analysis has been specifically designed with smoking as the pre‐specified primary exposure, using predefined eligibility criteria restricted to first‐time ablation procedures and exposure‐definition‐stratified analysis. Therefore, this study aims to systematically review the existing literature and perform a meta‐analysis to assess the association between active smoking and the risk of AF recurrence post‐ablation, providing novel insights to inform lifestyle modification strategies, with current evidence supporting smoking cessation as a potentially beneficial approach to improve ablation outcomes.

## 2 Method

### 2.1 Study Design

The present systematic review and meta‐analysis followed preferred reporting items for systematic reviews and meta‐analyses (PRISMA) reporting standards and methodological guidance from the Cochrane handbook for systematic reviews of interventions [21]. A protocol for this review was prospectively registered in PROSPERO (registration number: CRD420261278406).

### 2.2 Eligibility Criteria

Eligibility criteria were predefined using the PICO framework. The population included patients with atrial fibrillation undergoing a first catheter ablation procedure. The exposure of interest was smoking status, with never smokers serving as the reference group. The primary outcome was atrial fibrillation recurrence following catheter ablation. Studies were excluded if they were (1) secondary research, including review papers, meta‐analyses, editorials, and commentaries; (2) conference abstracts without full text; and (3) inaccessible full text.

### 2.3 Search Strategy and Selection Process

A systematic literature search was conducted across six databases, including PubMed, Scopus, the Cochrane Library, Epistemonikos, Scilit, and ProQuest, up to March 24, 2026. Search strategies were tailored to each database and incorporated relevant keywords and controlled vocabulary, aligned with the Medical Subject Headings (MeSH) in PubMed, as detailed in File S1. No restrictions were applied regarding publication year or language. All retrieved records were imported into Rayyan.ai for duplicate removal and screening [22]. Two reviewers (E.D.P.H. and A.M.J.T.) independently screened titles and abstracts and subsequently assessed the full texts of potentially eligible studies. Any disagreements were resolved through discussion with a third reviewer (F.P.S.W.) until consensus was reached.

### 2.4 Data Extraction

Data extraction was performed for all included studies using a standardized tabular format. Extracted items comprised study characteristics, including author, year of publication, study design, country, sample size, smoking status, age, sex distribution, body mass index (BMI), and mean follow‐up duration. Clinical data included atrial fibrillation subtype, ablation modality, left ventricular ejection fraction (LVEF), left atrial diameter (LAD), and relevant comorbidities, such as hypertension, diabetes mellitus, heart failure, coronary artery disease, prior stroke, hyperlipidemia, dyslipidemia, and alcohol consumption. The number of recurrence events among smokers and non‐smokers was also recorded. Two reviewers (F.P.S.W. and H.T.C.) extracted the data independently, and the extracted dataset was verified by a third reviewer (A.M.T.S.). Statistical analyses were conducted by F.P.S.W.

### 2.5 Quality Assessment and Certainty of Evidence

Risk of bias was evaluated using the Risk Of Bias In Non‐randomized Studies of Exposures (ROBINS‐E) tool, which is designed to assess non‐randomized studies of exposure effects across seven domains: confounding, participant selection, exposure classification, deviations from intended exposures, missing data, outcome measurement, and selection of the reported result [23]. Domain‐level judgments informed the overall risk‐of‐bias rating for each included study. Final judgments were tabulated and visualized as traffic‐light plots using ROBVIS [24].

The certainty of evidence for the primary outcome was assessed using the Grading of Recommendations Assessment, Development and Evaluation (GRADE) framework. Risk‐of‐bias judgments derived from the ROBINS‐E assessment were incorporated into the GRADE evaluation. The certainty of evidence was assessed across the domains of risk of bias, inconsistency, indirectness, imprecision, and publication bias and categorized as high, moderate, low, or very low. Two reviewers (M.R.A. and A.M.T.S.) independently performed the assessment of quality asessment and certainty of evidence, any disagreements were resolved through consensus with a third reviewer (F.P.S.W.).

### 2.6 Statistical Analysis

Statistical analyses were conducted in RStudio version 4.4.1 using the meta package. When continuous variables were reported as median and interquartile range, they were converted to mean and standard deviation using established estimation methods. For studies reporting data across multiple groups, pooled means and standard deviations were calculated according to Cochrane‐recommended formulas. Effect estimates were summarized as odds ratios (ORs) with 95% confidence intervals (CIs). Because only a small number of included studies reported adjusted hazard ratios, and these were subject to substantial heterogeneity in adjustment sets, a generic inverse‐variance synthesis of hazard ratios was not feasible; pooled odds ratios were therefore calculated from raw recurrence event counts among smokers and non‐smokers using a pairwise meta‐analysis approach. Pooled estimates were generated using a random‐effects model with restricted maximum likelihood estimation. Between‐study heterogeneity was quantified using the *I* ^2^ statistic, with values greater than 50% considered to indicate substantial heterogeneity [25]. Statistical significance was defined as a two‐sided *p*‐value < 0.05. Publication bias was examined visually using funnel plots and statistically using Egger's regression test. The Duval and Tweedie trim‐and‐fill method was applied to evaluate the potential influence of missing studies on the pooled estimate. Sensitivity analyses were performed to assess the stability of the findings, while subgroup and meta‐regression analyses were conducted to explore potential sources of heterogeneity.

## 3 Result

### 3.1 Study Selection

**FIGURE 1:**
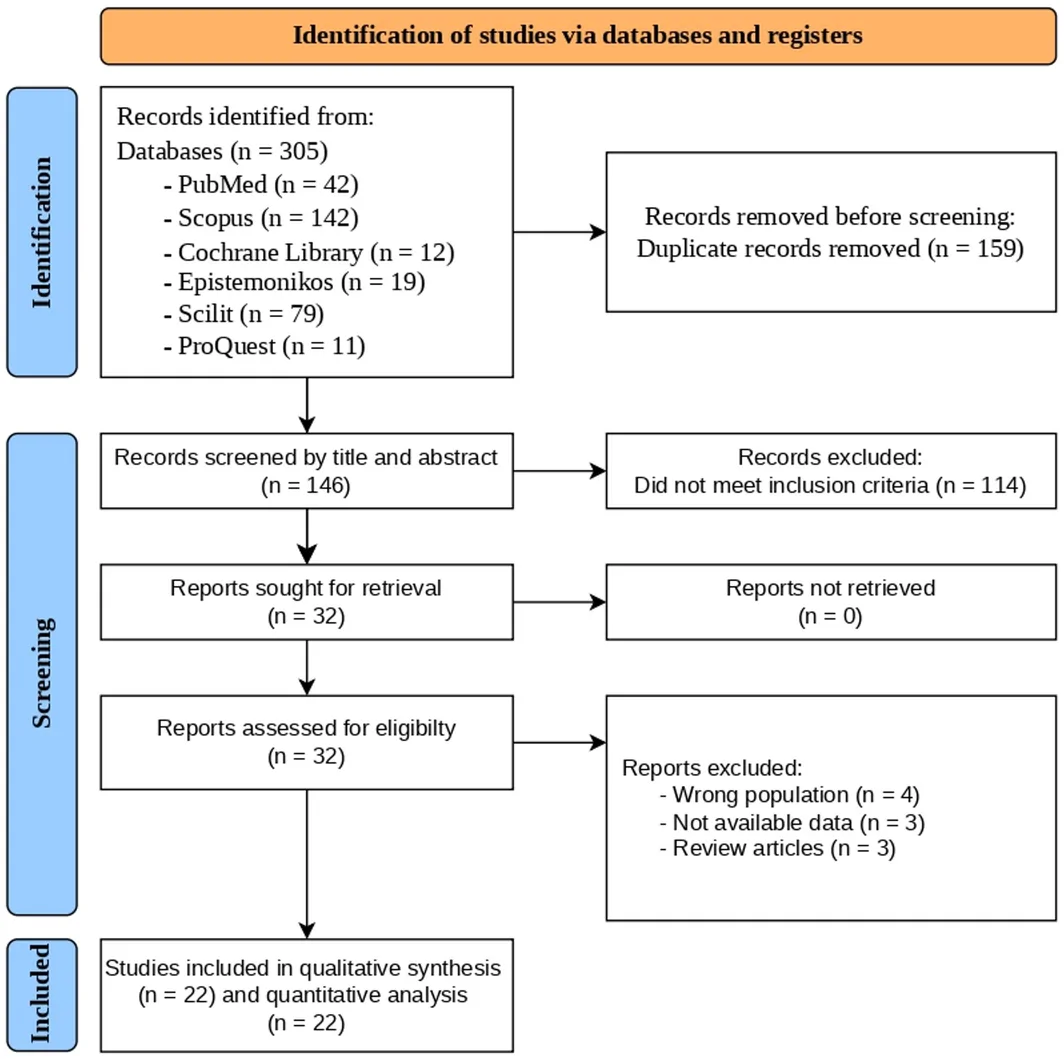
Study selection process.

A total of 305 records were retrieved from the six databases and 159 duplicates were removed. Of these, 146 records were screened by title and abstract and 114 were excluded for not meeting the eligibility criteria. No studies were excluded due to inability to retrieve full texts. Consequently, 32 studies were assessed for full‐text, of which 10 were excluded due to inappropriate population, unavailable data, or being review articles. Ultimately, 22 studies were included in this review (Figure 1).

### 3.2 Demographical Characteristic

A total of 6047 participants were included, comprising 3845 men and 2202 women. Smoking exposure across the included studies was defined as ever smoking (*n* = 9), current smoking (*n* = 4), or not explicitly stated (*n* = 9). The studies were conducted across multiple countries, including Austria, China, Egypt, Germany, Italy, Japan, Taiwan, and Turkey. The mean age of participants was 58.6 years, with a BMI of 25.5 kg/m^2^ and a mean follow‐up duration of 22.2 months. Detailed demographics are presented in Table 1.

**TABLE 1:** Demographic characteristic of included studies.

Author	Study design	Country	Sample size	Smoking Status	Age	Male/female	BMI	Mean follow‐up (month)
Allam et al. 2018 [26]	Prospective	Egypt	36	NA	44.3 ± 12.1	26/10	NA	NA
Aytemir et al. 2015 [27]	Prospective	Turkey	306	Ever smoker	55.3 ± 10.6	144/162	24.7 ± 3.1	23.0
Beyer et al. 2021 [28]	Retrospective	Austria	732	NA	57.8 ± 10.8	536/196	26.9 ± 4.0	31.0
Buiatti et al. 2016 [29]	Retrospective	Germany	76	NA	45.0 ± 8.0	53/23	26.0 ± 4.0	14.6
Cheng et al. 2018 [18]	Retrospective	Taiwan	201	Ever smoker	52.7 ± 9.7	177/24	NA	31.0
Cui et al. 2025 [30]	Prospective	China	345	Ever smoker	61.0 ± 2.6	227/118	26.7 ± 3.5	27.9
Dugral et al. 2022 [15]	Retrospective	Turkey	139	Ever smoker	57.0 ± 11.0	71/68	NA	36.7
Fukamizu et al. 2010 [31]	Prospective	Japan	59	Ever smoker	60.0 ± 11.0	48/11	NA	10.1
Giomi et al. 2024 [16]	Retrospective	Italy	186	Ever smoker	63.4 ± 9.7	135/51	26.5 ± 4.1	13.7
Gu et al. 2024 [32]	Prospective	China	53	NA	61.7 ± 3.5	28/25	26.7 ± 0.8	7.9
Huang et al. 2023 [33]	Retrospective	China	638	Current	65.8 ± 10.5	306/332	24.8 ± 3.2	15.1
Knappe et al. 2025 [34]	Prospective	Germany	132	NA	64.0 ± 5.1	79/53	26.3 ± 1.0	26.3
Ling et al. 2025 [35]	Prospective	China	145	NA	60.6 ± 11.7	90/55	24.2 ± 2.7	NA
Liu et al. 2024 [36]	Retrospective	Taiwan	638	Ever smoker	56.3 ± 11.2	472/166	25.1 ± 3.4	NA
Ma et al. 2025 [37]	Retrospective	China	827	Ever smoker	61.1 ± 11.8	549/278	24.8 ± 3.4	43.7
Okar et al. 2018 [38]	Prospective	Turkey	100	Current	55.2 ± 10.7	53/47	28.3 ± 4.6	NA
Su et al. 2019 [39]	Prospective	China	277	NA	65.5 ± 10.4	147/130	26.4 ± 2.9	14.2
Wang et al. 2019 [40]	Prospective	China	521	NA	60.2 ± 9.6	327/194	24.5 ± 4.2	NA
Yalcin et al. 2015 [41]	Prospective	Turkey	80	Current	54.3 ± 7.7	48/32	24.7 ± 3.8	15.3
Zhan et al. 2024 [42]	Prospective	China	100	Ever smoker	61.3 ± 6.2	53/47	23.8 ± 2.2	NA
Zhao et al. 2020 [43]	Prospective	China	304	Current	59.9 ± 9.8	182/122	24.7 ± 3.2	NA
Zhu et al. 2025 [44]	Retrospective	China	152	NA	66.1 ± 10.2	94/58	24.5 ± 3.1	NA

### 3.3 Clinical Characteristics

The clinical characteristics of the included patients comprised both paroxysmal and persistent atrial fibrillation. Catheter ablation techniques included cryoballoon, radiofrequency, and combined approaches (cryoballoon plus radiofrequency). Reported comorbidities included hypertension, diabetes mellitus, heart failure, coronary artery disease, prior stroke, hyperlipidemia, dyslipidemia, and alcohol consumption. The mean LVEF and LAD were 61.9% and 40.01 mm, respectively. Detailed clinical characteristics are provided in File S2. Among included studies, 8 studies performed PVI alone, while the remaining 14 incorporated an additional lesion set, most commonly linear ablation (roof, mitral isthmus, or cavotricuspid isthmus line), complex fractionated atrial electrogram (CFAE) ablation, or substrate zone modification, in particular for studies with persistent AF. However, only two studies [18, 31] reported the extent of additional ablation separately for smokers versus non‐smokers, while the remaining studies reported this information only at the overall cohort level without stratification by smoking status (File S3).

### 3.4 Quality Assessment

**FIGURE 2:**
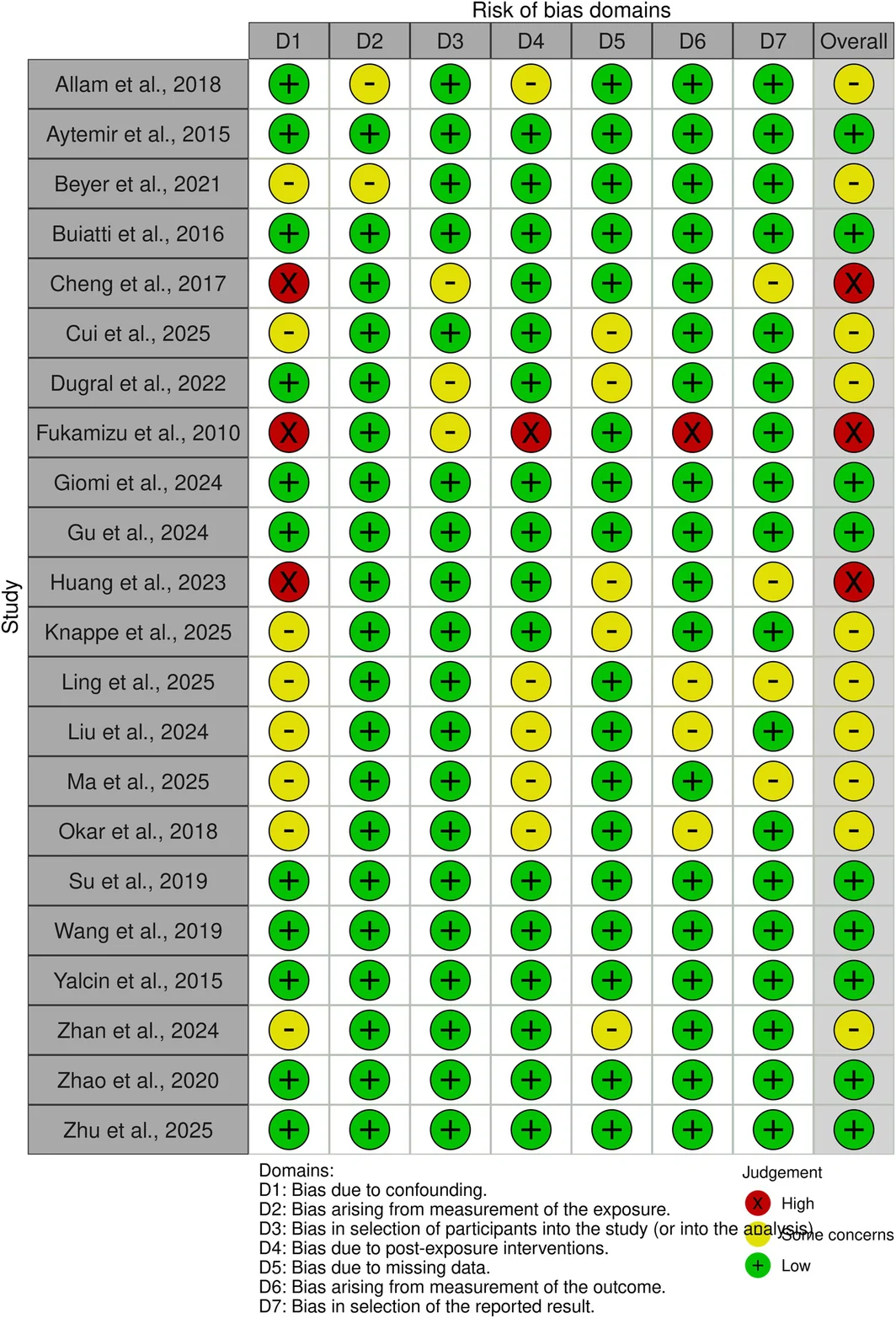
Risk of bias assessment.

The risk‐of‐bias assessment using ROBINS‐E is summarized in Figure 2. Overall, nine studies were judged as having low risk of bias, ten studies raised some concerns, and three studies were classified as high risk. Non‐low judgments were mainly driven by confounding and post‐exposure interventions. Concerns regarding confounding generally reflected incomplete adjustment for key prognostic factors, such as demographic characteristics, atrial fibrillation subtype, body mass index, left atrial size, comorbidities, ablation strategy, or follow‐up procedures. For post‐exposure interventions, some concerns or high‐risk judgments were assigned when post‐ablation management, including antiarrhythmic drug use, repeat ablation, cardioversion, lifestyle modification, or smoking cessation, was not clearly reported or controlled. Other domains contributed less substantially to the overall judgment, although several studies had unclear smoking definitions, incomplete reporting of follow‐up losses, heterogeneous recurrence monitoring, or limited information on prespecified outcome reporting. Overall, the risk‐of‐bias profile supports the inclusion of these studies, but residual confounding, inconsistent exposure definitions, and variation in post‐ablation management should be considered when interpreting the pooled estimates.

### 3.5 Association of Smoking Status and Risk of Atrial Fibrillation Recurrence

**FIGURE 3:**
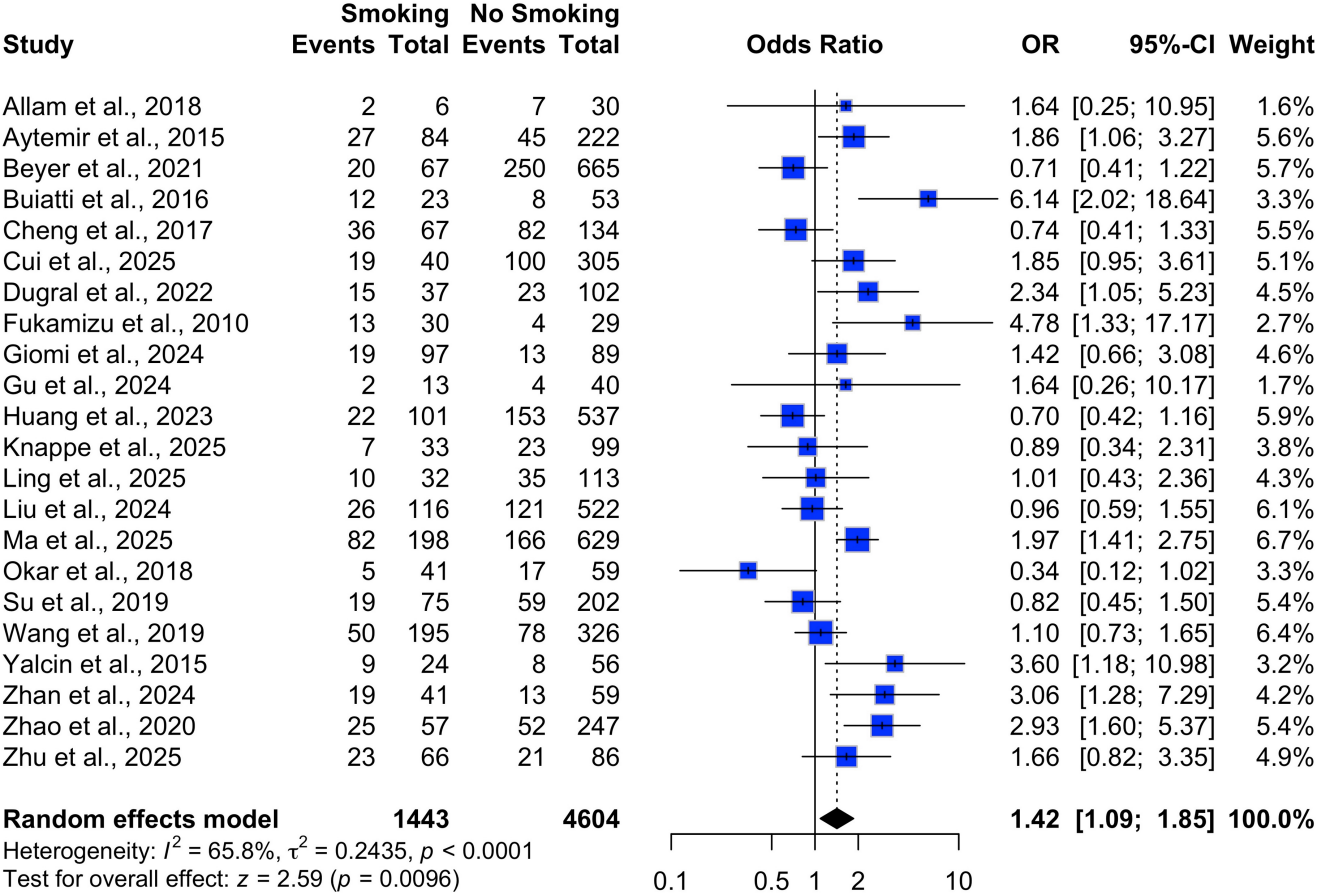
Smoking status and risk of atrial fibrillation recurrence after catheter ablation.

Pooled analysis demonstrated that smoking was associated with a significantly increased odds of AF recurrence (OR 1.42, 95% CI 1.09–1.85; *p* = 0.0096), with substantial heterogeneity (*I* ^2^ = 65.8%) (Figure 3). Sensitivity analysis using a leave‐one‐out approach showed that exclusion of the study by Buiatti et al. slightly reduced heterogeneity (*I* ^2^ = 63.1%) while maintaining a statistically significant association (OR 1.35, 95% CI 1.05–1.73; *p* = 0.0207), indicating robustness of the findings (File S4). Assessment of publication bias showed no significant, as indicated by Egger's test (*p* = 0.4280) (File S5). However, trim‐and‐fill analysis imputed two potentially missing studies, resulting in a slightly attenuated and non‐significant pooled estimate (OR 1.30, 95% CI 0.98–1.73; *p* = 0.0636), with persistent substantial heterogeneity (*I* ^2^ = 68.6%).

### 3.6 Subgroup and Meta Regression Analysis

**FIGURE 4:**
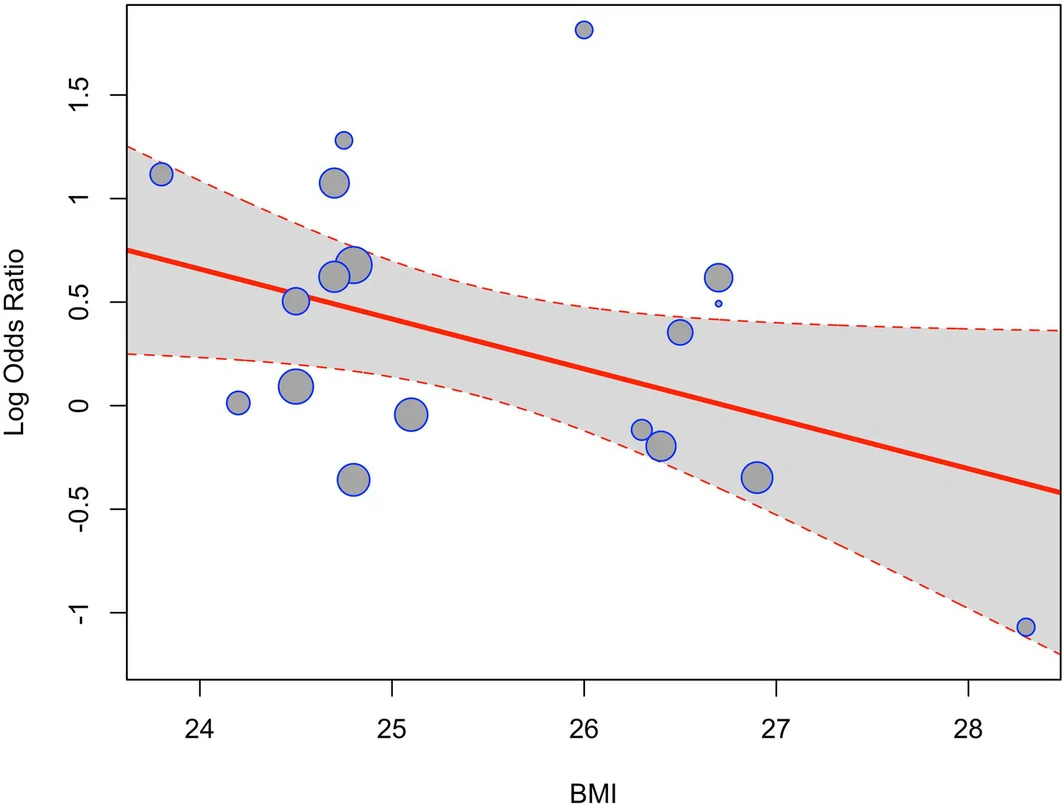
Meta‐regression analysis examining the effect of BMI on the association between smoking and atrial fibrillation recurrence.

The subgroup and meta‐regression analyses are presented in Table 2. Subgroup analysis by study design showed a significant association in prospective studies (OR 1.54, 95% CI 1.07–2.22; *p* = 0.0206), whereas retrospective studies did not reach statistical significance (OR 1.30, 95% CI 0.87–1.94; *p* = 0.1928), with no significant difference between subgroups (*p* for subgroup difference = 0.5471). By country, a significant association was observed in studies conducted in China (OR 1.46, 95% CI 1.04–2.03; *p* = 0.0277) and Japan (OR 4.78, 95% CI 1.33–17.17; *p* = 0.0165). Estimates from other regions were not statistically significant, including Austria (OR 0.71, 95% CI 0.41–1.22; *p* = 0.2124), Egypt (OR 1.64, 95% CI 0.25–10.95; *p* = 0.6079), Germany (OR 2.29, 95% CI 0.34–15.17; *p* = 0.3916), Italy (OR 1.42, 95% CI 0.66–3.08; *p* = 0.3700), Taiwan (OR 0.86, 95% CI 0.59–1.25; *p* = 0.4381), and Turkey (OR 1.57, 95% CI 0.63–3.92; *p* = 0.3375). The difference across countries was borderline (*p* for subgroup difference = 0.0637). Subgroup analysis by smoking status demonstrated a significant association in studies defining exposure as ever smoking (OR 1.66, 95% CI 1.20–2.29; *p* = 0.0023), while no significant association was observed in studies restricted to current smokers (OR 1.27, 95% CI 0.44–3.68; *p* = 0.6637) or in studies without a clearly reported definition (OR 1.17, 95% CI 0.82–1.69; *p* = 0.3899). No significant subgroup difference was detected (*p* for subgroup difference = 0.3734). Subgroup analysis based on ablation modality showed a significant association in studies using radiofrequency ablation (OR 1.55, 95% CI 1.13–2.14; *p* = 0.0068), whereas no significant associations were observed in cryoballoon studies (OR 1.34, 95% CI 0.37–4.94; *p* = 0.6558) or mixed approaches (OR 1.04, 95% CI 0.65–1.66; *p* = 0.8811). There was no significant difference between ablation subgroups (*p* for subgroup difference = 0.3804). Detailed forest plots for the subgroup analyses are provided in File S6. Meta‐regression analysis identified body mass index (BMI) as the only significant moderator (*β* = −0.2410, 95% CI −0.4806 to −0.0487; *p* = 0.0487; Figure 4), suggesting that the association between smoking and AF recurrence may be attenuated in populations with higher BMI.

**TABLE 2:** Subgroup and meta‐regression analysis examining factors influencing the association between smoking and atrial fibrillation recurrence.

Subgroup analysis					
Variable	Number of study	OR	95% CI	*p*	*p* sub. difference
**Study design**					0.5471
Prospective	13	1.54	1.07 to 2.22	0.0206*	
Retrospective	9	1.30	0.87 to 1.94	0.1928	
**Country**					0.0637
Austria	1	0.71	0.41 to 1.22	0.2124	
China	10	1.46	1.04 to 2.03	0.0277*	
Egypt	1	1.64	0.25 to 10.95	0.6079	
Germany	2	2.29	0.34 to 15.17	0.3916	
Italy	1	1.42	0.66 to 3.08	0.3700	
Japan	1	4.78	1.33 to 17.17	0.0165*	
Taiwan	2	0.86	0.59 to 1.25	0.4381	
Turkey	4	1.57	0.63 to 3.92	0.3375	
**Smoking status**					0.3734
Ever smoker	9	1.66	1.20 to 2.29	0.0023*	
Current smoker	4	1.27	0.44 to 3.68	0.6637	
Not reported	9	1.17	0.82 to 1.69	0.3899	
**Type of ablation**					0.3804
Cryoballon	3	1.34	0.37 to 4.94	0.6558	
Radiofrequency	15	1.55	1.13 to 2.14	0.0068*	
Mix	4	1.04	0.65 to 1.66	0.8811	

Meta‐regression				
Variable	Number of study	Estimate	95% CI	*p*
Mean age	22	−0.0304	−0.0837 to 0.0229	0.2642
Male	22	−0.0005	−0.0264 to 0.0254	0.9704
BMI	18	−0.2410	−0.4806 to −0.0487	0.0487*
Sample Size	22	−0.0008	−0.0019 to 0.0003	0.1350
Paroxysmal AF	21	0.0065	−0.0047 to 0.0178	0.2558
LVEF (%)	18	−0.0089	−0.0871 to 0.0694	0.8246
LAD (mm)	15	0.0030	−0.1261 to 0.1321	0.9636
Hypertension	20	−0.0184	−0.0421 to 0.0053	0.1274
Diabetes mellitus	21	0.0241	−0.0050 to 0.0532	0.1041
Heart failure	8	−0.0081	−0.0528 to 0.0365	0.7209
Coronary artery disease	12	0.0115	−0.0333 to 0.0563	0.6150
Stroke	10	0.0068	−0.0978 to 0.1114	0.8990
Hyperlipidemia	3	0.0251	−0.1817 to 0.2320	0.8117
Dyslipidemia	3	0.0210	−0.0123 to 0.0544	0.2167
Alcohol consumption	10	0.0025	−0.0173 to 0.0223	0.8019
Mean follow‐up time (month)	14	−0.0071	−0.0425 to 0.0284	0.6964

Abbreviations: AF, atrial fibrillation; LAD, left atrial diameter; LVEF, left ventricular ejection fraction.

* *p* < 0.05 statistically significant.

### 3.7 Certainty of Evidence

According to the GRADE framework, the overall certainty of evidence for the association between smoking and atrial fibrillation recurrence after catheter ablation was rated as low (Table 3). The certainty was downgraded because of concerns regarding residual confounding and variability across studies. No serious concerns were identified regarding indirectness or imprecision. Detailed GRADE assessments are presented in File S7.

**TABLE 3:** GRADE analysis.

No. of studies	Participants	Relative effect (95% CI)	Absolute effect	Certainty of evidence (GRADE)
22 observational studies	6047	OR 1.42 (1.09–1.85)	76 more recurrences per 1000 patients (18 more to 138 more)	⊕⊕◯◯ LOW

## 4 Discussion

Recurrence of AF was a pivotal landmark in rhythm control, in which episodes of recurrence may significantly increase the risk of major adverse cardiovascular events (MACE), especially thromboembolic events [45, 46]. In the present meta‐analysis, smoking was associated with a higher risk of AF recurrence after catheter ablation, although the observed effect was modest and graded as low‐certainty evidence. Meta‐regression identified BMI as the only significant moderator (*β* = −0.2410, 95% CI −0.4806 to −0.0487; *p* = 0.0487), indicating a weaker association at higher BMI. Other variables, including age, sex, sample size, comorbidities, alcohol consumption, and LVEF, were not significantly associated with the effect size.

The modality‐stratified pattern on subgroup analysis warrants cautious interpretation, since there was a non‐substantial *p*‐value for subgroup differences and should be regarded as hypothesis‐generating. One possible explanation is that radiofrequency delivery is more contact‐ and operator‐dependent, with lesion durability shaped by catheter stability, contact force, and local tissue characteristics, whereas cryoballoon ablation produces a more standardized, circumferential lesion set that is comparatively less contingent on operator technique and may therefore be less sensitive to underlying patient‐level risk factors [47]. However, the procedural covariates needed to test this hypothesis directly were incompletely reported. In 22 included studies, only Beyer et al. reported operator experience, describing ablation at a high‐volume center, and only two reported contact‐force‐guided delivery, which was Giomi et al. using the CLOSE protocol and very‐high‐power short duration, and Gu et al. reporting ablation index and contact‐force targets [16, 28, 32]. No formal first‐pass isolation rate metric stratified by smoking status is available across the 22 included studies. This apparent divergence may therefore reflect unmeasured differences in procedural platform, operator skill, and technology level variation. More broadly, because radiofrequency and cryoballoon cohorts in the included studies were not randomly allocated, the observed subgroup difference may equally reflect differences in study era, patient selection, or other unmeasured confounders, rather than a true interaction between smoking and ablation modality. Given the non‐significant *p*‐value for subgroup difference, this finding should not be interpreted as evidence of effect modification by ablation technique.

A further important consideration in interpreting our result was that AF recurrence after ablation not only depended on comorbidity, but also on procedural (PV isolation completeness) and underlying substrate. On this occasion, due to incomplete reporting across studies, our data could not be formally explored for these two factors, as mechanistic data stratified by smoking status were available in only two of the 22 included studies. Cheng et al. reported a higher prevalence of non‐pulmonary vein triggers among smokers, despite no significant difference in overall recurrence, and Fukamizu et al. reported PV reconnection findings together with left atrial volume data by smoking status [18, 31]. These findings support the interpretation that smoking may act as only one of the significant comorbidities in AF recurrence by contributing to an adverse atrial substrate, although this small base should be regarded as hypothesis‐generating.

A similar caveat applies to the geographical pattern observed in our analysis, with significant associations observed in studies from China and Japan, while other regions showed non‐significant results. Notably, the Japan estimate was derived from a single study with a wide confidence interval (95% CI 1.33–17.17) and should be interpreted with caution rather than as robust evidence of a region‐specific effect. The wide confidence intervals in several regions suggest limited statistical power rather than a true absence of association. Differences in smoking patterns, healthcare systems, and procedural practices may contribute, but the current data are insufficient to support strong region‐specific conclusions.

BMI emerged as the only significant moderator, with a negative association suggesting that the effect of smoking on recurrence may be attenuated in populations with higher BMI. This finding is consistent with prior evidence showing a complex relationship between smoking and body weight, where smoking is often associated with lower BMI [48]. One possible explanation is that obesity‐related atrial remodeling, including atrial enlargement and fibrosis, may have a dominant effect on recurrence risk, thereby reducing the relative contribution of smoking [49]. This supports the idea that AF recurrence is driven by multiple interacting risk factors rather than a single exposure [50]. Other variables, including age, sex, comorbidities, and left ventricular ejection fraction, were not significantly associated with the effect size in meta‐regression, suggesting that smoking may act independently of these factors, although this interpretation is limited by study‐level data. Individual‐level interactions cannot be fully assessed in this context, and residual confounding remains possible.

These results are broadly consistent with, yet complementary to previous literature. Chan et al., in a pooled meta‐analysis of risk factors for AF recurrence post‐ablation, similarly identified a smoking history as a risk factor of recurrence, drawing from pooled 1122 patients across three studies [20]. Our analysis, conducted on a larger and more recent cohort, reinforces and extends this earlier signal, further weighing smoking as a reproducible predictor of recurrence in AF ablation. Alongside other established predictors, Li et al. identified persistent AF, diabetes, left atrial enlargement, and inflammatory markers as key determinants of recurrence, highlighting the central role of atrial remodeling [51]. These convergent findings argue for incorporating smoking history alongside these more established, higher‐magnitude predictors within future risk‐stratification models for post‐ablation recurrence.

Definitional heterogeneity in smoking exposure is a further, consequential limitation. Included studies variably defined exposure as current smoking, ever smoking, or left status unspecified, with limited reporting of intensity, duration, or cessation timing. This is not only a semantic distinction because the category of ever smoking captures cumulative lifetime exposure, including both current and former smokers, and may therefore better reflect the long‐term biological effects of tobacco‐related atrial remodeling. In contrast, studies restricted to current smoking may be more susceptible to misclassification and heterogeneity in exposure definition, which can attenuate the observed association. This is consistent with broader evidence showing that smoking‐related cardiovascular risk is strongly influenced by cumulative exposure and declines only gradually after cessation [52].

Mechanistically, the observed association is plausible. Smoking contributes to oxidative stress, systemic inflammation, and atrial fibrosis, all of which promote structural and electrical remodeling of the atrium. These changes may facilitate the persistence of arrhythmogenic substrates even after pulmonary vein isolation [53]. In addition, smoking has been associated with an increased prevalence of non‐pulmonary vein triggers, which may reduce the long‐term durability of ablation and increase the likelihood of recurrence [18]. Evidence from epidemiological studies also supports a dose‐dependent relationship between smoking and AF risk, further reinforcing its role in arrhythmogenesis [54, 55].

This study has several strengths, including a relatively large pooled sample (6047 patients) and consistent findings across sensitivity analyses. However, several limitations should be acknowledged. Dose–response analysis could not be performed because smoking intensity was rarely reported across the included studies. Only Giomi et al. provided data related to smoking burden, such as pack‐years, while most other studies only classified participants as smokers or non‐smokers without further quantifying exposure intensity [16]. This limited reporting prevents assessment of whether higher cumulative smoking exposure is associated with a greater risk of AF recurrence after ablation. Therefore, future studies should report smoking intensity, duration, cessation timing, and post‐ablation smoking behavior to allow more detailed dose–response analyses. Moreover, substantial heterogeneity remained unexplained, smoking exposure was not uniformly defined, and meta‐regression was limited to study‐level data, which introduces potential ecological bias. In addition, some subgroup analyses were underpowered, and the attenuation of the effect after trim‐and‐fill analysis suggests that publication bias cannot be entirely excluded.

Future research should focus on individual patient‐level analyses to better evaluate dose–response relationships and interactions between smoking and other risk factors. Research on the effect of newer generations of single‐shot pulsed field ablation (PFA) is needed, as this platform potentially enables more PV durability with minimal operator and technique‐related variability that significantly impacts the likelihood of recurrence [56, 57]. Prospective studies that incorporate lifestyle modification, including smoking cessation, alongside catheter ablation may help clarify the clinical impact of these interventions on long‐term outcomes. Taken together, these priorities are consistent with the new 2024 AF‐CARE guideline paradigm, in which smoking is formally designated under the “C” domain, positioning smoking not merely as a trigger predisposing to new‐onset AF, but as an active driver of recurrence following successful ablation [58].

## 5 Conclusion

Smoking is associated with a modest increase in the risk of AF recurrence following catheter ablation. Although the effect size is smaller than that of established structural predictors, the association was consistent across analyses, supporting its clinical relevance. However, substantial heterogeneity and attenuation after adjustment for publication bias suggest the finding should be interpreted with caution. Future studies should focus on patient‐level data to assess smoking intensity, duration, and cessation, and explore interactions with other modifiable risk factors. Standardized definitions and prospective studies evaluating smoking cessation are also needed to clarify its clinical impact.

## Author Contributions

F.P.S.W., A.M.T.S., and R.P. contributed to the study conception and design. F.P.S.W., A.M.T.S, M.R.A., A.M.J.T., H.T.C., E.D.P.H., and R.P. performed the literature search, study selection, data extraction, and quality assessment. F.P.S.W. and A.A.Z.K. conducted the statistical analysis and interpreted the findings. F.P.S.W., A.M.T.S, M.R.A., A.A.Z.K., H.T.C., C.D.T., and R.P. drafted the manuscript. All authors critically revised the manuscript for important intellectual content and approved the final version for submission.

## Funding

The authors have nothing to report.

## Ethics Statement

Ethical approval and informed consent were not required because this study was a systematic review and meta‐analysis of previously published studies.

## Conflicts of Interest

The authors declare no conflicts of interest.

## Supporting information


**File S1:** Boolean operator.
**File S2:** Clinical characteristic.
**File S3:** Additional ablation beyond PVI in included studies.
**File S4:** Leave one out analysis.
**File S5:** Publication bias assessment using funnel plot (a), egger test (b), and trim and fill Duval and Tweedie (c).
**File S6:** Forest plot of subgroup analysis for study design (a), country (b), smoking status (c), and type of ablation.
**File S7:** Detailed of GRADE analysis.

## Data Availability

All data generated or analyzed during this study are included in this published article and its Supporting Information. Additional data are available from the corresponding author upon reasonable request.

## References

[1] S. Tan , Z. Zhang , Q. Lin , et al., “Burden and Age‐Specific Trends of Atrial Fibrillation/Atrial Flutter From 1990 to 2023: A Growing Challenge Among Younger and Middle‐Aged Adults,” Europace 28 (2026): euag036, 10.1093/europace/euag036. 41778603 PMC13034541

[2] Y. Yuniadi , A. I. Supit , D. A. Hanafy , et al., “Prevalence of Atrial Fibrillation Based on Tertiary Hospital Survey in Indonesia: A Smartphone‐Based Diagnosis,” Journal of Arrhythmia 40 (2024): 1102–1107, 10.1002/joa3.13137. 39416241 PMC11474569

[3] A. Buja , V. Rebba , L. Montecchio , et al., “The Cost of Atrial Fibrillation: A Systematic Review,” Value in Health 27 (2024): 527–541, 10.1016/j.jval.2023.12.015. 38296049

[4] J. P. Piccini , A. M. Russo , J. Peacock , et al., “Impact of Atrial Fibrillation Burden on Quality of Life and Prediction of Health Care Utilization: Primary Results From the DEFINE AFib Study,” Heart Rhythm 23 (2026): e1069–e1077, 10.1016/j.hrthm.2026.02.024. 41759867

[5] A. A. Razzack , H. M. Lak , S. Pothuru , et al., “Efficacy and Safety of Catheter Ablation vs Antiarrhythmic Drugs as Initial Therapy for Management of Symptomatic Paroxysmal Atrial Fibrillation: A Meta‐Analysis,” Reviews in Cardiovascular Medicine 23 (2022): 112, 10.31083/j.rcm2303112. 35345279

[6] R. A. Winkle , R. H. Mead , G. Engel , et al., “Very Long Term Outcomes of Atrial Fibrillation Ablation,” Heart Rhythm 20 (2023): 680–688, 10.1016/j.hrthm.2023.02.002. 36764350

[7] R. Vempati , A. Garg , M. Shah , et al., “Predictors of Atrial Fibrillation Recurrence After Catheter Ablation: A State‐Of‐The‐Art Review,” Heart 6 (2025): 12, 10.3390/hearts6020012.

[8] F. Liu , T. Song , Q. Hu , et al., “Body Mass Index and Atrial Fibrillation Recurrence Post Ablation: A Systematic Review and Dose‐Response Meta‐Analysis,” Frontiers in Cardiovascular Medicine 9 (2023): 999845, 10.3389/fcvm.2022.999845. 36818915 PMC9932032

[9] I. Anagnostopoulos , M. Kousta , C. Kossyvakis , et al., “Epicardial Adipose Tissue and Atrial Fibrillation Recurrence Following Catheter Ablation: A Systematic Review and Meta‐Analysis,” Journal of Clinical Medicine 12 (2023): 6369, 10.3390/jcm12196369. 37835012 PMC10573952

[10] A. Goette , U. Lendeckel , A. Kuchenbecker , et al., “Cigarette Smoking Induces Atrial Fibrosis in Humans via Nicotine,” Heart 93 (2007): 1056–1063, 10.1136/hrt.2005.087171. 17395670 PMC1955003

[11] R. B. Gonçalves , R. D. Coletta , K. G. Silvério , et al., “Impact of Smoking on Inflammation: Overview of Molecular Mechanisms,” Inflammation Research 60 (2011): 409–424, 10.1007/s00011-011-0308-7. 21298317

[12] O. Rom , K. Avezov , D. Aizenbud , and A. Z. Reznick , “Cigarette Smoking and Inflammation Revisited,” Respiratory Physiology & Neurobiology 187 (2013): 5–10, 10.1016/j.resp.2013.01.013. 23376061

[13] A. D'Alessandro , I. Boeckelmann , M. Hammwhöner , and A. Goette , “Nicotine, Cigarette Smoking and Cardiac Arrhythmia: An Overview,” European Journal of Preventive Cardiology 19 (2012): 297–305, 10.1177/1741826711411738. 22779085

[14] Y. S. Levitzky , C.‐Y. Guo , J. Rong , et al., “Relation of Smoking Status to a Panel of Inflammatory Markers: The Framingham Offspring,” Atherosclerosis 201 (2008): 217–224, 10.1016/j.atherosclerosis.2007.12.058. 18289552 PMC2783981

[15] E. Duğral , O. E. Turan , A. A. Başkurt , and E. E. Özcan , “Impact of Smoking on Long Term Atrial Fibrillation Ablation Success,” Journal of Basic and Clinical Health Sciences 6 (2022): 268–276, 10.30621/jbachs.1003047.

[16] A. Giomi , A. Bernardini , A. P. Perini , et al., “Clinical Impact of Smoking on Atrial Fibrillation Recurrence After Pulmonary Vein Isolation,” International Journal of Cardiology 413 (2024): 132342, 10.1016/j.ijcard.2024.132342. 38971534

[17] S. Li , J. Zhang , S. Zuo , et al., “Patterns of Postablation Recurrence and Adverse Cardiovascular Outcomes in Patients With Atrial Fibrillation,” Journal of the American Heart Association 14 (2025): e038832, 10.1161/JAHA.124.038832. 40247625 PMC12184226

[18] W. Cheng , L. Lo , Y. Lin , et al., “Cigarette Smoking Causes a Worse Long‐Term Outcome in Persistent Atrial Fibrillation Following Catheter Ablation,” Journal of Cardiovascular Electrophysiology 29 (2018): 699–706, 10.1111/jce.13451. 29424013

[19] J. A. Joglar , M. K. Chung , A. L. Armbruster , et al., “2023 ACC/AHA/ACCP/HRS Guideline for the Diagnosis and Management of Atrial Fibrillation: A Report of the American College of Cardiology/American Heart Association Joint Committee on Clinical Practice Guidelines,” Circulation 149 (2024): e167, 10.1161/CIR.0000000000001193. 38033089 PMC11095842

[20] K. W. P. Chan , D. S. F. Yu , J. W. K. Ho , C. W. Y. Wong , and P. W. C. Li , “Identification of Clinical Risk Factors for Arrhythmia Recurrence in Patients With Atrial Fibrillation Undergoing Catheter Ablation: A Systematic Review and Meta‐Analysis,” Heart Rhythm (2026): S1547‐5271(26)02313–1, 10.1016/j.hrthm.2026.04.046. 42066879

[21] M. J. Page , J. E. McKenzie , P. M. Bossuyt , et al., “The PRISMA 2020 Statement: An Updated Guideline for Reporting Systematic Reviews,” BMJ 372 (2021): n71, 10.1136/bmj.n71. 33782057 PMC8005924

[22] M. Ouzzani , H. Hammady , Z. Fedorowicz , and A. Elmagarmid , “Rayyan—A Web and Mobile App for Systematic Reviews,” Systematic Reviews 5 (2016): 210, 10.1186/s13643-016-0384-4. 27919275 PMC5139140

[23] J. P. T. Higgins , R. L. Morgan , A. A. Rooney , et al., “A Tool to Assess Risk of Bias in Non‐Randomized Follow‐Up Studies of Exposure Effects (ROBINS‐E),” Environment International 186 (2024): 108602, 10.1016/j.envint.2024.108602. 38555664 PMC11098530

[24] L. A. McGuinness and J. P. T. Higgins , “Risk‐Of‐Bias VISualization (Robvis): An R Package and Shiny Web App for Visualizing Risk‐Of‐Bias Assessments,” Research Synthesis Methods 12 (2021): 55–61, 10.1002/jrsm.1411. 32336025

[25] J. Higgins , J. Thomas , J. Chandler , M. Cumpston , T. Li , and M. Page , “Cochrane Handbook for Systematic Reviews of Interventions Version 6.5 (Updated August 2024),” Cochrane (2024).

[26] L. E. Allam , A. M. A. E. Moteleb , and M. T. Ghanem , “Predictors of Short and Long Term Recurrences of Paroxysmal AF After Radiofrequency Ablation. Is Blanking Period Really Benign?,” Journal of Atrial Fibrillation 11 (2018): 2012, 10.4022/jafib.2012. 31139279 PMC6533841

[27] K. Aytemir , K. M. Gurses , M. U. Yalcin , et al., “Safety and Efficacy Outcomes in Patients Undergoing Pulmonary Vein Isolation With Second‐Generation Cryoballoon,” Europace 17 (2015): 379–387, 10.1093/europace/euu273. 25376699

[28] C. Beyer , L. Tokarska , M. Stühlinger , et al., “Structural Cardiac Remodeling in Atrial Fibrillation,” JACC: Cardiovascular Imaging 14 (2021): 2199–2208, 10.1016/j.jcmg.2021.04.027. 34147453

[29] A. Buiatti , B. Kaess , T. Reents , et al., “Catheter Ablation for “Lone” Atrial Fibrillation: Efficacy and Predictors of Recurrence,” Journal of Cardiovascular Electrophysiology 27 (2016): 536–541, 10.1111/jce.12936. 26799683

[30] N. Cui , H. Li , W. Sun , et al., “Clinical Utility of the 4S‐AF Scheme in Predicting Atrial Fibrillation Recurrence After Radiofrequency Catheter Ablation,” Reviews in Cardiovascular Medicine 26 (2025): 26318, 10.31083/RCM26318. 40160573 PMC11951279

[31] S. Fukamizu , H. Sakurada , M. Takano , et al., “Effect of Cigarette Smoking on the Risk of Atrial Fibrillation Recurrence After Pulmonary Vein Isolation,” Journal of Arrhythmia 26 (2010): 21–29, 10.1016/S1880-4276(10)80032-8.

[32] Y. Gu , H. Wang , G. Xue , et al., “Left Atrial Electrophysiological Properties After Pulmonary Vein Isolation Predict the Recurrence of Atrial Fibrillation: A Cohort Study,” Reviews in Cardiovascular Medicine 25 (2024): 167, 10.31083/j.rcm2505167. 39076500 PMC11267206

[33] W. Huang , H. Sun , Y. Tang , Y. Luo , and H. Liu , “Platelet‐To‐Lymphocyte Ratio Improves the Predictive Ability of the Risk Score for Atrial Fibrillation Recurrence After Radiofrequency Ablation,” Journal of Inflammation Research 16 (2023): 6023–6038, 10.2147/JIR.S440722. 38107387 PMC10723594

[34] D. Knappe , J. Vogler , J. Weimann , et al., “Left Atrial Reservoir Strain and Recurrence of Atrial Fibrillation Following De‐Novo Pulmonary Vein Isolation ― Results of the ASTRA‐AF Pilot Study,” Circulation Journal 89 (2025): 153–161, 10.1253/circj.CJ-24-0209. 38839350

[35] X. Ling , J. Li , J. Wu , et al., “Predictive Value of Echocardiographic Parameter of Diastolic Dysfunction (Ea/aa) Combined With Electrocardiographic P‐Wave Dispersion for the Detection of Early Recurrence of Atrial Fibrillation After Radiofrequency Catheter Ablation,” Frontiers in Cardiovascular Medicine 12 (2025): 1585919, 10.3389/fcvm.2025.1585919. 40951832 PMC12424079

[36] C.‐M. Liu , W.‐S. Chen , S.‐L. Chang , et al., “Use of Artificial Intelligence and I‐Score for Prediction of Recurrence Before Catheter Ablation of Atrial Fibrillation,” International Journal of Cardiology 402 (2024): 131851, 10.1016/j.ijcard.2024.131851. 38360099

[37] J. Ma , C. Wang , J. He , P. Zhang , Y. Pingzhen , and X. Li , “H‐Type Hypertension and U‐Shaped Body Mass Index Predict Atrial Fibrillation Recurrence After Catheter Ablation: Insights From a 3‐Year Follow‐Up,” Heart Rhythm O2 6 (2025): 1278–1288, 10.1016/j.hroo.2025.06.021. 41280537 PMC12635739

[38] S. Okar , O. Kaypakli , D. Y. Şahin , and M. Koç , “Fibrosis Marker Soluble ST2 Predicts Atrial Fibrillation Recurrence After Cryoballoon Catheter Ablation of Nonvalvular Paroxysmal Atrial Fibrillation,” Korean Circulation Journal 48 (2018): 920–929, 10.4070/kcj.2018.0047. 30238709 PMC6158454

[39] C. Su , Z. Liu , Y. Gao , et al., “Study on the Relationship Between Telomere Length Changes and Recurrence of Atrial Fibrillation After Radiofrequency Catheter Ablation,” Journal of Cardiovascular Electrophysiology 30 (2019): 1117–1124, 10.1111/jce.13958. 31042327

[40] Q. Wang , C. Zhuo , Y. Shang , et al., “U‐Shaped Relationship Between Left Atrium Size on Echocardiography and 1‐Year Recurrence of Atrial Fibrillation After Radiofrequency Catheter Ablation ― Prognostic Value Study ―,” Circulation Journal 83 (2019): 1463–1471, 10.1253/circj.CJ-19-0167. 31178525

[41] M. U. Yalcin , K. M. Gurses , D. Kocyigit , et al., “Cardiac Autoantibody Levels Predict Recurrence Following Cryoballoon‐Based Pulmonary Vein Isolation in Paroxysmal Atrial Fibrillation Patients,” Journal of Cardiovascular Electrophysiology 26 (2015): 615–621, 10.1111/jce.12665. 25788224

[42] J. Zhan , C. Peng , Y. Liu , et al., “Predictive Value of Serum microRNA‐29b‐3p in Recurrence of Atrial Fibrillation After Radiofrequency Catheter Ablation,” Clinical Interventions in Aging 19 (2024): 715–725, 10.2147/CIA.S450292. 38716143 PMC11075679

[43] J. Zhao , D. Zhou , M. Chen , et al., “CHA2DS2‐VASc and SAMe‐TT2R2 Scores as Predictors of Recurrence for Nonvalvular Atrial Fibrillation Patients on Vitamin K Antagonists After Radiofrequency Catheter Ablation,” Journal of Cardiovascular Medicine 21 (2020): 200–208, 10.2459/JCM.0000000000000930. 31977539

[44] Y. Zhu , M. Miao , Z. Xia , et al., “Association of HARMS2‐AF Score With Atrial Tachyarrhythmia Recurrence After Radiofrequency Catheter Ablation: A Retrospective Observational Study,” BMC Cardiovascular Disorders 25 (2025): 636, 10.1186/s12872-025-05110-y. 40877811 PMC12392465

[45] P. Kirchhof , A. J. Camm , A. Goette , et al., “Early Rhythm‐Control Therapy in Patients With Atrial Fibrillation,” New England Journal of Medicine 383 (2020): 1305–1316, 10.1056/NEJMoa2019422. 32865375

[46] R. Pranata , C. D. Tristan , E. Yonas , et al., “Early Catheter Ablation for Atrial Fibrillation After Acute Decompensated Heart Failure: A Systematic Review and Meta‐Analysis With Reconstructed Kaplan‐Meier Individual Patient Data,” Hellenic Journal of Cardiology (2026): S1109966626001326, 10.1016/j.hjc.2026.07.006. 42486366

[47] G. Kiełbasa and M. Jastrzębski , “Cryoballoon Pulmonary Vein Isolation as a Standard Approach for Interventional Treatment of Atrial Fibrillation. A Review and a Practical Guide to an Effective and Safe Procedure,” Advances in Interventional Cardiology 16 (2020): 359–375, 10.5114/aic.2020.101760. 33598008 PMC7863834

[48] M. Jacobs , “Adolescent Smoking: The Relationship Between Cigarette Consumption and BMI,” Addictive Behaviors Reports 9 (2019): 100153, 10.1016/j.abrep.2018.100153. 31193813 PMC6542372

[49] H. Shu , J. Cheng , N. Li , et al., “Obesity and Atrial Fibrillation: A Narrative Review From Arrhythmogenic Mechanisms to Clinical Significance,” Cardiovascular Diabetology 22 (2023): 192, 10.1186/s12933-023-01913-5. 37516824 PMC10387211

[50] X. Guo and J. Li , “Risk and Protective Factors of Recurrence After Catheter Ablation for Atrial Fibrillation,” Reviews in Cardiovascular Medicine 25 (2024): 81, 10.31083/j.rcm2503081. 39076956 PMC11263837

[51] G. Li , Y. Zhao , Z. Peng , and Y. Zhao , “Risk Factors for the Recurrence of Atrial Fibrillation After Catheter Ablation: A Meta‐Analysis,” Egyptian Heart Journal 77 (2025): 9, 10.1186/s43044-025-00605-7. PMC11729607 39804412

[52] M. Rahman , M. Alatiqi , M. Al Jarallah , et al., “Cardiovascular Effects of Smoking and Smoking Cessation: A 2024 Update,” Global Heart 20 (2025): 15, 10.5334/gh.1399. 39991592 PMC11843939

[53] P. Korantzopoulos , K. P. Letsas , G. Tse , N. Fragakis , C. A. Goudis , and T. Liu , “Inflammation and Atrial Fibrillation: A Comprehensive Review,” Journal of Arrhythmia 34 (2018): 394–401, 10.1002/joa3.12077. 30167010 PMC6111477

[54] D. Aune , S. Schlesinger , T. Norat , and E. Riboli , “Tobacco Smoking and the Risk of Atrial Fibrillation: A Systematic Review and Meta‐Analysis of Prospective Studies,” European Journal of Preventive Cardiology 25 (2018): 1437–1451, 10.1177/2047487318780435. 29996680

[55] W. Zhu , P. Yuan , Y. Shen , R. Wan , and K. Hong , “Association of Smoking With the Risk of Incident Atrial Fibrillation: A Meta‐Analysis of Prospective Studies,” International Journal of Cardiology 218 (2016): 259–266, 10.1016/j.ijcard.2016.05.013. 27236125

[56] M. Iqbal , W. Kamarullah , R. Pranata , et al., “Meta‐Analysis of Pulsed Field Ablation Versus Thermal Ablation for Pulmonary Vein Isolation in AF: A Broad Overview Focusing on Efficacy, Safety and Outcomes,” Arrhythmia & Electrophysiology Review 13, no. e13 (2024): e13, 10.15420/aer.2024.05. 39221061 PMC11363063

[57] I. Irnizarifka , C. D. Tristan , E. A. Budiono , et al., “Feasibility of Pulsed Field Ablation for Ventricular Arrhythmia: A Systematic Review,” Future Cardiology (2026): 1–13, 10.1080/14796678.2026.2699788. PMC13540101 42434806

[58] I. C. Van Gelder , M. Rienstra , K. V. Bunting , et al., “2024 ESC Guidelines for the Management of Atrial Fibrillation Developed in Collaboration With the European Association for Cardio‐Thoracic Surgery (EACTS),” European Heart Journal 45 (2024): 3314–3414, 10.1093/eurheartj/ehae176. 39210723

