# Supplementary material for: Smoking and Risk of Atrial Fibrillation Recurrence After Catheter Ablation: A Meta‐Analysis and Meta‐Regression
Source: J Arrhythm. 2026 Aug 24;42(4):e70452. doi: 10.1002/joa3.70452 (PMC13502057; doi:10.1002/joa3.70452)
Supplement: Supplementary file 1 [file JOA3-42-e70452-s001.docx]

**Supplementary Material**

**Supplementary File 1.** Boolean operator

| **Databases** | **Keywords combination** | **Hits** |
| --- | --- | --- |
| PubMed | (((((("Atrial Fibrillation*"[Title/Abstract]) OR (AF[Title/Abstract])) OR ("Auricular Fibrillation*"[Title/Abstract])) OR ("Atrial Fibrillation"[MeSH Terms])) AND (((((("Catheter Ablation"[Title/Abstract]) OR (ablation[Title/Abstract])) OR ("radiofrequency ablation"[Title/Abstract])) OR ("pulmonary vein isolation"[Title/Abstract])) OR (PVI[Title/Abstract])) OR ("Catheter Ablation"[MeSH Terms]))) AND (((((recurrence*[Title/Abstract]) OR (relapse[Title/Abstract])) OR ("treatment failure"[Title/Abstract])) OR (Recrudescence*[Title/Abstract])) OR (recurrence[MeSH Terms]))) AND (((Smoking[Title/Abstract]) OR (smoker*[Title/Abstract])) OR (Smoking[MeSH Terms])) | 42 |
| Scopus | ( TITLE-ABS-KEY ( "Atrial Fibrillation*" OR AF OR "Auricular Fibrillation*" ) AND TITLE-ABS-KEY ( "Catheter Ablation" OR ablation OR "radiofrequency ablation" OR "pulmonary vein isolation" OR PVI ) AND TITLE-ABS-KEY ( recurrence* OR relapse OR "treatment failure" OR Recrudescence* ) AND TITLE-ABS-KEY ( Smoking OR smoker* ) ) | 142 |
| Cochrane Library | ("Atrial Fibrillation" OR "Atrial Fibrillations" OR AF OR "Auricular Fibrillation" OR "Auricular Fibrillations"):ti,ab,kw AND ("Catheter Ablation" OR ablation OR "radiofrequency ablation" OR "pulmonary vein isolation" OR PVI):ti,ab,kw AND (recurrence* OR relapse OR "treatment failure" OR Recrudescence*):ti,ab,kw AND (Smoking OR smoker*):ti,ab,kw | 12 |
| Epistemonikos | title:("Atrial Fibrillation*" OR AF OR "Auricular Fibrillation*") OR abstract:("Atrial Fibrillation*" OR AF OR "Auricular Fibrillation*")) AND (title:("Catheter Ablation" OR ablation OR "radiofrequency ablation" OR "pulmonary vein isolation" OR PVI) OR abstract:("Catheter Ablation" OR ablation OR "radiofrequency ablation" OR "pulmonary vein isolation" OR PVI)) AND (title:(recurrence* OR relapse OR "treatment failure" OR Recrudescence*) OR abstract:(recurrence* OR relapse OR "treatment failure" OR Recrudescence*)) AND (title:(Smoking OR smoker*) OR abstract:(Smoking OR smoker*))) OR abstract:((title:("Atrial Fibrillation*" OR AF OR "Auricular Fibrillation*") OR abstract:("Atrial Fibrillation*" OR AF OR "Auricular Fibrillation*")) AND (title:("Catheter Ablation" OR ablation OR "radiofrequency ablation" OR "pulmonary vein isolation" OR PVI) OR abstract:("Catheter Ablation" OR ablation OR "radiofrequency ablation" OR "pulmonary vein isolation" OR PVI)) AND (title:(recurrence* OR relapse OR "treatment failure" OR Recrudescence*) OR abstract:(recurrence* OR relapse OR "treatment failure" OR Recrudescence*)) AND (title:(Smoking OR smoker*) OR abstract:(Smoking OR smoker*)))) | 19 |
| Scilit | ( Title, Abstract, Keyword ( "Atrial Fibrillation*" OR AF OR "Auricular Fibrillation*" ) AND Title, Abstract, Keyword ( "Catheter Ablation" OR ablation OR "radiofrequency ablation" OR "pulmonary vein isolation" OR PVI ) AND Title, Abstract, Keyword ( recurrence* OR relapse OR "treatment failure" OR Recrudescence* ) AND Title, Abstract, Keyword ( Smoking OR smoker* ) ) | 79 |
| ProQuest | abstract("Atrial Fibrillation*" OR AF OR "Auricular Fibrillation*") AND abstract("Catheter Ablation" OR ablation OR "radiofrequency ablation" OR "pulmonary vein isolation" OR PVI) AND abstract(recurrence* OR relapse OR "treatment failure" OR Recrudescence*) AND abstract(Smoking OR smoker*) | 11 |

**Supplementary File 2.** Clinical characteristic

| **Author** | **Type of AF** | **Type of ablation** | **LVEF (%)** | **LAD (mm)** | **Hypertension** | **DM** | **HF** | **CAD** | **Stroke** | **Hyperlipidemia** | **Dyslipidemia** | **Alcohol consumption** |
| --- | --- | --- | --- | --- | --- | --- | --- | --- | --- | --- | --- | --- |
| Allam et al., 2018 | Paroxysmal | Radiofrequency | 65.5 ± 3.0 | 42.3 ± 4.8 | 19 (52.8) | 5 (13.9) | NA | NA | NA | NA | NA | NA |
| Aytemir et al., 2015 | Paroxysmal: 245 (80.1)  Persistent: 59 (19.3) | Cryoballoon | 64.5 ± 5.0 | 38.9 ± 3.9 | 128 (41.8) | 41 (13.4) | NA | 34 (11.1) | NA | 74 (24.2) | NA | 15 (4.9) |
| Beyer et al., 2021 | Paroxysmal: 647 (88.4)  Persistent: 85 (11.6) | Radiofrequency: 270 (36.9)  Cryoballon: 462 (63.1) | NA | NA | 338 (46.2) | 30 (4.1) | NA | NA | NA | NA | 158 (21.6) | NA |
| Buiatti et al., 2016 | Paroxysmal: 62 (82)  Persistent: 14 (18) | Radiofrequency | NA | 37 ± 4 | NA | NA | NA | NA | NA | NA | NA | NA |
| Cheng et al., 2017 | Persistent | Radiofrequency | NA | NA | 101 (50.2) | 23 (11.4) | 42 (20.9) | 38 (19) | 14 (7) | 49 (24.4) | NA | NA |
| Cui et al., 2025 | Paroxysmal: 244 (70.7)  Persistent: 101 (29.2) | Radiofrequency | 62 ± 1.2 | 40 ± 1 | 212 (61.4) | 66 (19.1) | 136 (39.4) | 58 (16.8) | 30 (8.7) | NA | NA | 46 (13.3) |
| Dugral et al., 2022 | Paroxysmal: 109 (78.4)  Persistent: 30 (21.6) | Radiofrequency: 88 (63.3)  Cryoballon: 51 (36.7) | 57.3 ± 4.6 | 49.3 ± 6 | 65 (46.8) | 20 (14.4) | NA | NA | NA | NA | NA | NA |
| Fukamizu et al., 2010 | Paroxysmal: 39 (66)  Persistent: 20 (34) | Radiofrequency | 63 ± 15 | 38 ± 7 | 18 (31) | 11 (19) | 4 (7) | NA | 5 (8) | NA | NA | NA |
| Giomi et al., 2024 | Paroxysmal: 148 (79.6)  Persistent: 38 (20.4) | Radiofrequency | 57.4 ± 5.5 | NA | 105 (56.5) | 13 (7) | 19 (10.2) | 19 (10.2) | 5 (2.7) | NA | 85 (45.7) | 7 (3.8) |
| Gu et al., 2024 | Paroxysmal: 33 (62.3)  Persistent: 20 (37.7) | Radiofrequency | 65 ± 2.2 | 38.2 ± 1.8 | 32 (60.4) | 11 (20.8) | 7 (13.2) | 27 (50.9) | NA | NA | NA | 6 (11.3) |
| Huang et al., 2023 | Paroxysmal: 302 (47.3)  Persistent: 336 (52.7) | Radiofrequency | 59.0 ± 5.9 | 41.8 ± 5.4 | 349 (54.7) | 134 (21.0) | 132 (20.7) | 71 (11.1) | 34 (5.3) | NA | NA | NA |
| Knappe et al., 2025 | Paroxysmal: 88 (66.7)  Persistent: 44 (33.3) | Radiofrequency: 73 (55.3)  Cryoballon: 59 (44.7) | NA | NA | 78 (59.1) | 7 (5.3) | NA | 15 (11.4) | 13 (9.8) | NA | 18 (12.9) | NA |
| Ling et al., 2025 | Paroxysmal: 61 (42.1)  Persistent: 84 (57.9) | Radiofrequency | 62.7 ± 9.5 | NA | NA | 22 (15.2) | NA | 25 (17.2) | NA | NA | NA | NA |
| Liu et al., 2024 | Paroxysmal | Radiofrequency: 544 (85.3)  Cryoballon: 94 (14.7) | 57.9 ± 5.9 | 38.9 ± 5.7 | 251 (39.3) | 54 (8.5) | 25 (3.9) | 132 (20.7) | 14 (2.2) | 107 (16.8) | NA | NA |
| Ma et al., 2025 | Paroxysmal: 631 (76.3)  Persistent: 196 (23.7) | Radiofrequency | 56.8 ± 7.6 | NA | 442 (53.4) | 181 (21.9) | NA | NA | NA | NA | NA | 163 (19.7) |
| Okar et al., 2018 | Paroxysmal | Cryoballoon | 63.5 ± 7.7 | 36.5 ± 3.8 | 47 (47) | 13 (13) | NA | 9 (9) | NA | NA | NA | NA |
| Su et al., 2019 | Paroxysmal: 180 (65)  Persistent: 97 (35) | Radiofrequency | 64.4 ± 7.8 | 40.9 ± 5.5 | 172 (62.1) | 74 (26.7) | NA | 66 (23.8) | 34 (12.3) | NA | NA | NA |
| Wang et al., 2019 | Paroxysmal: 445 (85.4)  Persistent: 76 (14.6) | Radiofrequency | 68.0 ± 4.5 | 40.0 ± 6.0 | 237 (45.5) | 43 (8.3) | NA | NA | NA | NA | NA | 157 (30.1) |
| Yalcin et al., 2015 | Paroxysmal | Cryoballoon | 65.16 ± 3.20 | NA | 10 (12.5) | 17 (21.3) | NA | NA | NA | NA | NA | 9 (11.3) |
| Zhan et al., 2024 | NA | Radiofrequency | 58.4 ± 4.4 | 39.8 ± 3.3 | 48 (48) | 42 (42) | NA | NA | NA | NA | NA | 57 (57) |
| Zhao et al., 2020 | Paroxysmal: 256 (84.2)  Persistent: 48 (15.8) | Radiofrequency | 64.4 ± 6.9 | 39 ± 6 | 121 (39.8) | 28 (9.2) | 28 (9.2) | 57 (18.8) | 17 (5.6) | NA | NA | 62 (20.4) |
| Zhu et al., 2025 | Paroxysmal: 98 (64.5)  Persistent: 54 (35.5) | Radiofrequency | 58.7 ± 7.2 | 39.5 ± 5.7 | 104 (68.4) | 32 (21.1) | NA | NA | 20 (13.2) | NA | NA | 36 (23.7) |

Data are presented as mean ± standard deviation (SD) or n (%). Abbreviations: AF, atrial fibrillation; LVEF, left ventricular ejection fraction; LAD, left atrial diameter; DM, diabetes mellitus; CAD, coronary artery disease.

**Supplementary File 3.** Additional ablation beyond PVI in included studies.

| Study | PVI only / PVI+ | Additional set (type) | Reported by smoking status? (Y/N) | N smokers with additional set | N non-smokers with additional set |
| --- | --- | --- | --- | --- | --- |
| Allam et al., 2018 | PVI only | None needed | N (N/A) | Not reported by smoking status | Not reported by smoking status |
| Aytemir et al., 2015 | PVI only | None | N (N/A) | Not reported by smoking status | Not reported by smoking status |
| Beyer et al., 2021 | PVI only | None | N (N/A) | Not reported by smoking status | Not reported by smoking status |
| Buiatti et al., 2016 | PVI+ | CTI (paroxysmal); Stepwise (CFAE, linear lesions) for persistent lone AF | N | Not reported by smoking status | Not reported by smoking status |
| Cheng et al., 2018 | PVI+ | Linear (roof, mitral isthmus) and CFAE | Y | Linear: 47; CFAE: 31 | Linear: 89; CFAE: 78 |
| Cui et al., 2025 | PVI+ | CTI, LA linear, or low-voltage zone ablation | N | Not reported by smoking status | Not reported by smoking status |
| Dugral et al., 2022 | PVI only | None | N (N/A) | Not reported by smoking status | Not reported by smoking status |
| Fukamizu et al., 2010 | PVI+ | Routine CTI; CFAE or mitral isthmus modification | Y | 7 | 3 |
| Giomi et al., 2024 | PVI+ | Roof, mitral isthmus, or CTI lines for persistent AF | N | Not reported by smoking status | Not reported by smoking status |
| Gu et al., 2024 | PVI only | None | N (N/A) | Not reported by smoking status | Not reported by smoking status |
| Huang et al., 2023 | PVI+ | Low-voltage substrate modification for nonparoxysmal AF | N | Not reported by smoking status | Not reported by smoking status |
| Knappe et al., 2025 | PVI only | None (Index) | N (N/A) | Not applicable | Not applicable |
| Ling et al., 2025 | PVI+ | Roof, posterior, mitral/tricuspid isthmus, SVC isolation | N | Not reported by smoking status | Not reported by smoking status |
| Liu et al., 2024 | PVI+ | Linear and substrate modification as needed | N | Not reported by smoking status | Not reported by smoking status |
| Ma et al., 2025 | PVI+ | CTI block and left atrial lines | N | Not reported by smoking status | Not reported by smoking status |
| Okar et al., 2018 | PVI only | None | N (N/A) | Not reported by smoking status | Not reported by smoking status |
| Su et al., 2019 | PVI+ | Linear (roof, mitral, CTI) and CFAE | N | Not reported by smoking status | Not reported by smoking status |
| Wang et al., 2019 | PVI+ | SVC, LA roof, and CTI lines | N | Not reported by smoking status | Not reported by smoking status |
| Yalcin et al., 2015 | PVI only | None | N (N/A) | Not reported by smoking status | Not reported by smoking status |
| Zhan et al., 2024 | PVI+ | Linear and fragmentation potential ablation | N | Not reported by smoking status | Not reported by smoking status |
| Zhao et al., 2020 | PVI+ | SVC, LA linear, and CTI ablation | N | Not reported by smoking status | Not reported by smoking status |
| Zhu et al., 2025 | PVI+ | Roof, bottom, mitral, CTI lines | N | Not reported by smoking status | Not reported by smoking status |
| PVI, pulmonary vein isolation; CTI, cavotricuspid isthmus; CFAE, complex fractionated atrial electrogram; LA, left atrium; SVC, superior vena cava; N/A, not applicable. "PVI only" indicates studies in which pulmonary vein isolation was the sole ablation strategy performed, with no additional lesion sets. "PVI+" indicates studies in which one or more adjunctive ablation sets were performed in addition to PVI, as specified in the "Additional set (type)" column | | | | | |

**Supplementary File 4.** Leave one out analysis

**Supplementary File 5.** Publication bias assessment using funnel plot (a), egger test (b), and trim and fill Duval and Tweedie (c).

(a)

(b)

(c)

**Supplementary File 6.** Forest plot of subgroup analysis for study design (a), country (b), smoking status (c), and type of ablation

(a)

(b)

(c)

(d)

**Supplementary File 7.** Detailed of GRADE analysis

| **Certainty assessment** | | | | | | | **№ of patients** | | **Effect** | | **Certainty** | **Importance** |
| --- | --- | --- | --- | --- | --- | --- | --- | --- | --- | --- | --- | --- |
| **No of studies** | **Study design** | **Risk of bias** | **Inconsistency** | **Indirectness** | **Imprecision** | **Other considerations** | **Smoking** | **No Smoking** | **Relative (95% CI)** | **Absolute (95% CI)** |  |  |
| **AF recurrence (follow-up: range 7.9 months to 36.7 months)** | | | | | | | | | | | | |
| 22 | non-randomised studies | serious^a^ | serious^b^ | not serious | not serious | none | 458/1443 (31.7%) | 1282/4604 (27.8%) | **OR 1.42** (1.09 to 1.85) | **76 more per 1,000** (from 18 more to 138 more) | ⨁⨁◯◯ Low^a,b^ | CRITICAL |

**CI:** confidence interval; **OR:** odds ratio

#### **Explanations**

a. Most studies were judged as having some concerns or high risk of bias, primarily due to residual confounding and inadequate control of post-ablation interventions, which may influence the association between smoking and AF recurrence.

b. Downgraded one level for inconsistency due to substantial unexplained heterogeneity across studies (I² = 65.8%), which persisted after sensitivity analyses.
